# Supplementary material for: MAIVeSS: streamlined selection of antigenically matched, high-yield viruses for seasonal influenza vaccine production
Source: Nat Commun. 2024 Feb 6;15:1128. doi: 10.1038/s41467-024-45145-x (PMC10847134; doi:10.1038/s41467-024-45145-x)
Supplement: Supplementary file 1 — Supplementary Information [file 41467_2024_45145_MOESM1_ESM.pdf]

Supplementary Information

Title: MAIVeSS: Streamlined selection of antigenically matched, high-yield viruses for seasonal influenza vaccine production

Running title: Machine-learning assisted rapid selection of influenza vaccine seeds

Author list

Cheng Gao[1,2,3,4,¶], Feng Wen[5¶], Minhui Guan[1,3,4], Bijaya Hatuwal[1,2,3,4], Lei Li[6,7], Beatriz Praena[1,3,4], Cynthia Y. Tang[1,3,4,8], Jieze Zhang[9], Feng Luo[10], Hang Xie[11], Richard Webby[12] Yizhi Jane Tao[13], and Xiu-Feng Wan[1,2,3,4,5,8*]

Affiliations

[1]Center for Influenza and Emerging Infectious Diseases, University of Missouri, Columbia, MO 65211, USA; [2]Department of Electrical Engineering & Computer Science, College of Engineering, University of Missouri, Columbia, MO 65211, USA;
[3]Department of Molecular Microbiology and Immunology, School of Medicine, University of Missouri, Columbia, MO 65211, USA;
[4]Bond Life Sciences Center, University of Missouri, Columbia, MO 65211, USA;
[5]Department of Basic Sciences, College of Veterinary Medicine, Mississippi State University, MS 39762, USA; [6]Department of Chemistry, Georgia State University, Atlanta, GA 30303, USA;
[7]Center for Diagnostics & Therapeutics, Georgia State University, Atlanta, GA 30303, USA;
[8]Institute for Data Science and Informatics, University of Missouri, Columbia, MO 65211, USA;
[9]Department of Bioengineering, Rice University, Houston, TX, 77030 USA;
[10]University School of Computing, Clemson University, Clemson, SC 29634, USA;
[11]Laboratory of Respiratory Viral Diseases, Division of Viral Products, Office of Vaccines Research and Review, Center for Biologics Evaluation and Research, US Food and Drug Administration, Silver Spring, MD 20993, USA;
[12]Department of Infectious Diseases, St. Jude Children's Research Hospital, Memphis, TN 63141, USA;
[13]Department of BioSciences, Rice University, Houston, TX 77251, USA.

*Corresponding Author: Dr. Xiu-Feng Wan, Phone: (573)882-8943. E-mail: wanx@missouri.edu

[¶]These authors contributed equally.

1. Supplementary Information

1.1. Supplementary Data.

Validation of glycan binding profile for D131E-S193T-A198S using biolayer interferometry analyses
To confirm the binding avidities observed in the glycan microarray, we used biolayer interferometry to analyze the binding of the HY^{both} mutant D131E-S193T-A198S to five representative glycan analogs: Neu5Acα2-3Galβ1-4GlcNAcβ (3′SLN))(Lectinity Holdings, Moscow, Russia; catalog number 0036-BP), Neu5Acα2-6Galβ1-4GlcNAcβ (6′SLN)(Lectinity Holdings, Moscow, Russia; catalog number 0997-BP), Neu5Acα2-3Galβ1-4[Fucα1-3]GlcNAcβ (sLe^X), Neu5Gcα2-3Galβ1-4GlcNAcβ (3′SLN(Gc)), and Neu5Gcα2-3Galβ1-4[Fucα1-3]GlcNAcβ ($sLe^{X(Gc)}$). The HY^{both} mutant had broadened binding avidities from 6′SLN to 3′SLN and sLe^X whereas WT CA/04 did not bind to 3′SLN and sLe^X. The mutant had a 1.61-fold lower binding avidity to 6′SLN than to 3′SLN, and it did not bind to 3′SLN(Gc) or $sLe^{X(Gc)}$, similar to WT CA/04. These results were consistent with those obtained from the glycan microarray.

Genomic sequencing
Viral RNA was isolated from 200 µl of the sample using a 5X MagMAXTM Pathogen RNA/DNA kit (Thermo Fisher Scientific, Pittsburgh, PA; catalog number 4462359) according to the manufacturer's instructions, and a total of 80 µl RNA was obtained. cDNA synthesis was carried out using SuperScript III Reverse Transcriptase (Invitrogen, Grand Island, NY; catalog number 18080044) with 10 µl of the isolated RNA and the influenza virus-specific primer Uni12 (5'-AGCAAAAGCAGG-3'), with a total reaction volume of 25 µl. The HA segment of mutants was amplified using the Phusion High-Fidelity PCR Kit (Thermo Fisher Scientific, Pittsburgh, PA; catalog number F553L) and the primers CA/04_HA_F (5'-ATGAAGGCAATACTAGTAGTTCTGC-3') and CA/04_HA_R (5'-TTAAATACATATTCTACACTGTAGAGACC-3'). The PCR products (50 µl) were purified using the GeneJET PCR Purification kit (Thermo Fisher Scientific, Pittsburgh, PA; catalog number K0702) as per the manufacturer's instructions. The HA sequences of the mutants were confirmed by Sanger sequencing.

Generation of ferret antisera
Ferret antisera were produced in male or female ferrets aged 6 to 8 weeks, which were confirmed to be seronegative for CA/04, A/Switzerland/9715293/2013 (H3N2), and A/Hong Kong/4801/2014(H3N2). Each ferret was intranasally inoculated with 10^6 TCID50 of either the wild-type virus or a mutant virus to be tested. Ferret sera were collected 21 days after inoculation and used for antigenic phenotyping through serological assays.

Model comparison and parameter optimization
To ensure the robustness of our analyses, we compared the performance of our sparse learning model with three other commonly used sparse models: LASSO, RIDGE, and SGL. Additionally, we also compared our model with two other sparse learning methods, the L1- and L2-norm regularized method and the L1- and L∞-norm Composite Absolute Penalties method (iCAP). LASSO uses L1-norm regularization[1], RIDGE uses L2-norm regularization[2], SGL uses group Lasso regularization[3], L1- and L2-norm regularization combines L1-norm and L2-norm regularization[4], and iCAP combines L1-norm and L∞-norm regularization[5]. The performance of these models was evaluated based on various metrics, such as accuracy, Root Mean Square Error (RMSE), and predictive power. Briefly, the LASSO regression seeks to minimize the following:

$$||y - Xw||_2 + \lambda_1 ||w||_1, \tag{1}$$

the RIDGE regression seeks to minimize the following:

$$||y - Xw||_2 + \lambda_1||w||_2, \quad (2)$$

the RIDGE regression seeks to minimize the following:

$$||y - Xw||_2 + \lambda_1 \sum_l ||w_l||_2 + \lambda_2||w||_1, \quad (3)$$

the L1- and L2-norm regularized method seeks to minimize:

$$||y - XW||_2 + \lambda_1||[||W_{G_1}||_{\gamma_1}, ||W_{G_2}||_{\gamma_2}, \cdots, ||W_{G_n}||_{\gamma_n}, \cdots]||_{\gamma_0}, \quad (4)$$

and the iCAP seeks to minimize:

$$||y - XW||_2 + \lambda_1||[||W_{G_1}||_{\gamma_1}, ||W_{G_2}||_{\gamma_2}, \cdots, ||W_{G_n}||_{\gamma_n}, \cdots]||_{\gamma_0}, \quad (5)$$

where y is the vector of actual response value, w is the vector of weights, X is the matrix of explanatory value, λ_1 is constraint parameters, and $|| \cdot ||1$ is the L1-norm, $|| \cdot ||2$ is the L2-norm, G_n's, n = 1, · · · , N is indices of *n*-th pre-defined group, W_{G_n} is corresponding vector of weight, $||\cdot||\gamma_n$is group norm, and $||\cdot||\gamma_0$ is overall norm. Here we chose γ_0 equals 1 as the overall norm. If we choose that $\gamma_1 = \gamma_2 = \cdots = \gamma_N = \infty$ as group norm, which we refer to as the algorithm iCAP. If we choose that $\gamma_1 = \gamma_2 = \cdots = \gamma_N = 2$ as group norm, which we refer to the L1- and L2-norm regularized method. In addition, our comparison included three primary influenza antigenicity-related machine learning models mentioned in the literature, along with three deep learning approaches. The conventional machine learning methods consist of Support Vector Machine (SVM),[6, 7] [8], Naïve Bayes,[9-12] and Random Forest.[13-16] The deep learning methods include Gated Recurrent Unit (GRU), Long Short-Term Memory (LSTM), and Transformer.

For antigenicity analyses experiments, we set c = 1e-1 for LASSO, c = 5e3 for RIDGE, c_1 = 1e2 and c_2 = 3e1 for SGL, c = 9e2 for L1- and L2-norm regularized method, and c = 1e4 for iCAP. We used "fitcsvm" function in MATLAB with default settings for SVM, applied function "binaryBayesianLogReg" function for Naïve Nayes with K = 1, and set number of trees as 8 and depth as 2 for Random Forest. We created an LSTM network that consists of an LSTM layer with 50 hidden units, followed by a fully connected layer of size 10 and a dropout layer with dropout probability 0.5. We trained the entire model with the Adam optimizer and set the mini-batches size as 20. The initial learning rate is 2e-4. We created a GRU network that consists of a GRU layer with 50 hidden units, followed by a fully connected layer of size 10 and a dropout layer with dropout probability 0.5. We trained the entire model with the Adam optimizer and set the mini-batches size as 20. The initial learning rate is 2e-4. We also created a classification head network by setting the sequence input layer with 90 hidden units, followed by a fully connected layer of size 10 and a dropout layer with dropout probability 0.5. We trained the entire model with the Adam optimizer and set the mini-batches size as 20. The initial learning rate is 2e-4. For yield experiments, we set c = 1e-2 for LASSO, c = 1 for RIDGE, c_1 = 1e-1 and c_2 = 1e-1 for SGL, c = 1e-1 for L1- and L2-norm regularized method, c = 1e-1 for iCAP. For glycan binding experiments, we set c = 1e-1 for LASSO, c = 1e-1 for RIDGE, c_1 = 1e2 and c_2 = 3e1 for SGL, c = 9e2 for L1- and L2-norm regularized method, c = 1e4 for iCAP.

The PIMA (Protein-Protein Interactions in Macromolecular Analysis) method was utilized to incorporate the biochemical properties of amino acids. PIMA assigns the 20 amino acids into nine groups and assigns a different numerical code for different mutations. Substitutions between different pairs of residues are given an inclusive weight between 0 and 5. The weights assigned to each feature in the learning results indicate the significance of the feature, with greater weight indicating higher significance.

To investigate the impact of amino acid substitutions on growth phenotype, we utilized a three-group method for assigning amino acids to different groups based on their biophysical properties. Specifically, we classified each amino acid into one of three groups: nonpolar (including V, L, I, M, C, F, W, and Y), small nonpolar (including G, A, and P), and polar/charged (including S, T, N, Q, H, D, E, K, and R) [17]. Using this classification, if a mutation occurred between two groups at a given residue *j* (e.g., nonpolar to small polar), we set the j-th element of the feature vector x_i to 1; otherwise, we set it to 0. This approach allowed us to evaluate the directionality of amino acid substitutions on the growth phenotype.

The regularization parameters of the sparse learning model were optimized using root mean square error (RMSE), as shown in Fig. S4 to S9. The choice of regularization methods (LASSO, RIDGE, SGL, L1- and L2-norm, or iCAP) and the scoring method were also based on RMSE, determined through 10-fold cross-validation. In this method, 90% of the data were used for training and 10% for testing, and the model's performance was evaluated based on the RMSE, with smaller values indicating better performance. RMSE was defined as:

$$\mathrm{RMSE} = \sqrt{\frac{1}{n}\sum_{i=1}^{n}(\hat{y}_i - y_i)^2}\,, \tag{6}$$

To evaluate the performance of our method, we conducted a comparison based on accuracy. Specifically, we considered a threshold of 4-fold (2 units of antigenic distance) to determine if two viruses were antigenically distinct and exhibited antigenic drift. Using this threshold, we defined classification tasks to measure the prediction accuracy. The accuracy metric describes the proportion of correctly predicted results among the total number of samples.

Performance matrices

The performance matrices consist of accuracy, specificity/recall, sensitivity, precision, and F1 score. We computed both the training performance from 10-fold cross-validation (90% data for model development) and the testing performance (10% split data not used in model development).

$$Accuracy = \frac{number\ of\ correct\ predictions}{total\ number\ of\ predictions}, \tag{7}$$

$$Sensitivity = \frac{True\ positive}{True\ positive + False\ nageative}, \tag{8}$$

$$Specificity = \frac{True\ nagetive}{False\ nagetive + True\ nagative}, \tag{9}$$

$$Precision = \frac{True\ positive}{True\ positive + False\ positive}, \tag{10}$$

$$F1\ score = \frac{2 * Precision * Sensitivity}{Precision + Sensitivity}, \tag{11}$$

Bootstrapping analyses

To evaluate the reliability of the selected features by MTL-GGSL, we conducted 100 independent experiments with 80% of the training data in each experiment. We only retained features with a bootstrap value of at least 80 across multiple tasks, resulting in a set of unique features that were chosen as the final features learned by MTL-GGSL.

1.2. Discussion

Related machine learning methods

Over the past few years, several computational models have been developed to identify influenza antigenic variants using genomic sequences. These models include sparse learning[18-21], bivariate correlation analysis,[22] Bayesian model,[23, 24] naïve Bayes classifier,[9-12] random forest ,[13] regression models,[21, 25-28] decision tree algorithms,[29] and convolutional neural network model.[30, 31] Among these models, sparse learning has proven to be efficient and generalizable in identifying the association between residues and antigenicity of multiple subtypes of IAVs, including H1N1, H3N2, and H5N1[19-21, 32]. Additionally, generalized hierarchical sparse models have been used to identify the synergistic effects of multiple amino acid substitutions on antigenic changes.[33] To overcome the challenges in data integration, multi-task machine learning was developed, which assigns datasets to individual tasks and considers the relationship between different tasks[34]. In another study, group Least Absolute Shrinkage and Selection Operator (LASSO) was developed to accommodate multiple types of features and explore the relationships between different feature groups[32]. Although these models have proven effective in identifying antigenic variants, none of them have considered virus yield. Therefore, they cannot be used to directly identify antigenic match and high-yield viruses that can be produced readily based on genetic sequences.

Recently, a deep learning model, so called Machine Learning-guided Antigenic Evolution Prediction (MLAEP) was reported to predict antigenic evolution of SARS-CoV-2 viruses.[35] MLAEP applies transformer network to model the binding affinities between SARS-CoV-2 Receptor Binding Domain (RBD) and eight monoclonal RBD specific antibody and between RBD and human ACE2 receptor proteins. The genetic algorithm was used to generate synthetic RBD variants, and the binding affinities to those antibodies are evaluated by the model, which will identify those with potential mutant escape while maintaining high ACE2 binding ability. This study brought a novel concept on how to integrate both structure and sequences for machine learning. However, as a deep learning model, the inherent features associated with model performance are not intuitive and interpretable. Nevertheless, MLAEP has an advantage in its utilization of a transformer network to systematically integrate structural information, which MAIVeSS currently lacks. Although we have already incorporated amino acid proximity derived from structures into our learning process, we intend to adopt a similar strategy from MLAEP in our future studies.

Features associated with egg adapted amino acid changes in literature

Amino acid changes Y/H17Y, A57V, D131E, R193S, L194I, V223M, D225D/G, Q226R, and G228G/A in 2009 H1N1 viruses were identified when comparing cell-derived and egg-derived isolates.[36] These residues are believed to indicate potential egg adaptation. Among them, D127E, L191I, D225G/N, and Q226R can influence the antigenic properties.[36] From the 30 residues we identified as being associated with the antigenic properties of the 2009 H1N1 viruses, only position 225 has been noted in literature as a potential egg-adapted substitution (Table 1). Concerning residues linked to virus growth in either eggs or cells for 2009 H1N1 viruses, positions 131, 193, and 225 are reported as likely egg-adapted substitutions (Table 2).

List of Supplementary Figures

Supplementary Figure 1. Characteristics of the HA RBS mutants. A) The number of mutant viruses with at least one amino acid substitution located in the HA antibody binding sites of H1N1 viruses; B) the number of amino acid substitutions for each mutant in the HA RBS mutant library; C) biophysical properties of amino acid substitutions in the HA RBS mutants. The amino acids were grouped in three categories: nonpolar (including V, L, I, M, C, F, W, and Y) in light blue, small nonpolar (including G, A, and P) in pink, and polar/charged (including S, T, N, Q, H, D, E, K, and R) in light green. Source data are provided as a Source Data file.

Supplementary Figure 2. The relative yields in eggs (y-axis) and in cells (x-axis) of 196 HA RBS mutants compared to the CA/04 WT virus. Source data are provided as a Source Data file.

Supplementary Figure 3. List of 75 glycoforms printed on the glycan microarray.

Supplementary Figure 4. Heat map illustration of binding intensity of viruses to glycans on the glycan microarray. Each row represents a HA RBS mutant and each column represents an individual glycan. The color bar represents the magnitude of the binding intensity. The linkage hierarchical clustering was performed by Hierarchical Clustering Explorer 3.0 (https://www.cs.umd.edu/hcil/multi-cluster/) to generate the heat map and tree structure. Source data are provided as a Source Data file.

Supplementary Figure 5. List of 27 glycan substructures used in machine learning. These 27 substructures are separated into three groups: terminal, internal, or basal substructures.

Supplementary Figure 6. Glycan binding specificity of the HA D131E-S193T-A198S mutant by Bio-Layer Interferometry analyses. Binding of viruses to five representative glycan analogs, [Neu5Acα2-3Galβ1-4GlcNAcβ (3′SLN), Neu5Acα2-6Galβ1-4GlcNAcβ (6′SLN), and Neu5Acα2-3Galβ1-4[Fucα1-3]GlcNAcβ (sLe^{X}), Neu5Gcα2-3Galβ1-4GlcNAcβ (3′SLN(Gc)), and Neu5Gcα2-3Galβ1-4[Fucα1-3]GlcNAcβ (sLe$^{X(Gc)}$)], were determined by Bio-Layer Interferometry (Pall ForteBio LLC, Fremont, CA). The streptavidin-coated biosensors were first preloaded with biotin-labeled sialic acid receptors, followed by the 1 pM each virus binding for 1,200 seconds in a standard kinetic buffer with neuraminidase inhibitors (zanamivir hydrate and oseltamivir phosphate). Sialic acid receptor concentrations were titrated to 1 μg/ml (3′SLN and 6′SLN) or 1uM (sLeX) when loading with the biotin-labeled receptors. The binding response unit (nm) was recorded at the 1,196 second time point (4 seconds before the start of dissociation).

Supplementary Figure 7. Structural modeling showing the synergistic effects of amino acid substitution N159K, K166Q and S206T on the binding affinity of HA to SA2-3Gal. The results showed that residue 166 interacts with an alpha-helix at the C-terminal end of the 130-loop and the K166Q substitution may affect the conformation of the 130-loop, resulting in changes in binding affinity towards glycan receptors. In addition, residue 206 is located at the HA inter-subunit interface adjacent to the 220-loop, and the S206T substitution could potentially alter the structural conformation of the 220-loop, thus affecting the glycan receptor binding. The three-dimensional structure of HA protein was modeled based on the crystal structure of CA/04 HA in complex with 6′SLN (PDB ID# 3UBN) and 3′SLN (PDB ID# 3UBQ). Coot was first used to introduce the desired mutation to the three subunits of a HA trimer (*51*). The mutated coordinates were subsequently refined by energy minimization using Phenix (*52*). Structure figures were made using Pymol (The PyMOL Molecular Graphics System, Version 1.3, Schrödinger, LLC).

Supplementary Figure 8. Optimization of the parameters λ_1, λ_2, and λ_3 used in multi-task feature learning of MTL-GGSL for antigenicity analyses. Source data are provided as a Source Data file.

Supplementary Figure 9. Optimization of parameters λ used in GHSM for in cell and egg yield analyses. Source data are provided as a Source Data file.

Supplementary Figure 10. Optimization of the parameters λ_1, λ_2, and λ_3 in multi-task feature learning of MTL-GGSL for glycan binding analyses in the HY^{both} group. Source data are provided as a Source Data file.

Supplementary Figure 11. Optimization of the parameters λ_1, λ_2, and λ_3 in multi-task feature learning of MTL-GGSL for glycan binding analyses in the HY^{cell} group. Source data are provided as a Source Data file.

Supplementary Figure 12. Optimization of the parameters λ_1, λ_2, and λ_3 in multi-task feature learning of MTL-GGSL for glycan binding analyses in the HY^{egg} group. Source data are provided as a Source Data file.

Supplementary Figure 13. Optimization of the parameters λ_1, λ_2, and λ_3 in multi-task feature learning of MTL-GGSL for glycan binding analyses in the LY^{both} group. Source data are provided as a Source Data file.

Supplementary Figure 14. Correlation between viral titration TCID50 in and the total proteins obtained from ultracentrifugation purification of supernatants from virus-infected cell or egg cultures. Pearson correlation analysis was conducted using Prism. Typically, a coefficient r between 0.4 and 0.7 is considered to indicate a moderate positive correlation, while a value above 0.7 signifies a strong positive correlation. Source data are provided as a Source Data file.

Supplementary Figure 15. Quantification of HA proteins from egg-grown A/California/04/2009(HA, NA)×A/Puerto Rico/8/1934(H1N1). Ultracentrifugation products totaling 5 ug were treated with 500 units of PNGase F, followed by analysis using 12% SDS-PAGE. The intensities quantified by Image J are shown in the right table for five major proteins components (NP, NA, HA1, M, and HA2) identified in the SDS gel, and the HA1 and HA2 consist of 40.01% of the total proteins. Source data are provided as Supplementary Figure 16.

Supplementary Figure 16. Uncropped scans of blots showing the quantification of HA proteins from egg-grown A/California/04/2009(HA, NA)×A/Puerto Rico/8/1934(H1N1). The results presented in Supplementary Figure 15 are highlighted within the red box. Sample loading information for each lane is as follows: Lane 1: BSA (1 mg/ml); Lane 2: Marker; Lane 3: 5 ug virus treated with 500 units of PNGase F; Lane 4: 50 ug virus treated with 500 units of PNGase F; Lane 5: BSA (0.03 mg/ml); Lane 6: BSA (0.0625 mg/ml); Lane 7: BSA (0.125 mg/ml); Lane 8: BSA (0.25 mg/ml); Lane 9: BSA (0.5 mg/ml).

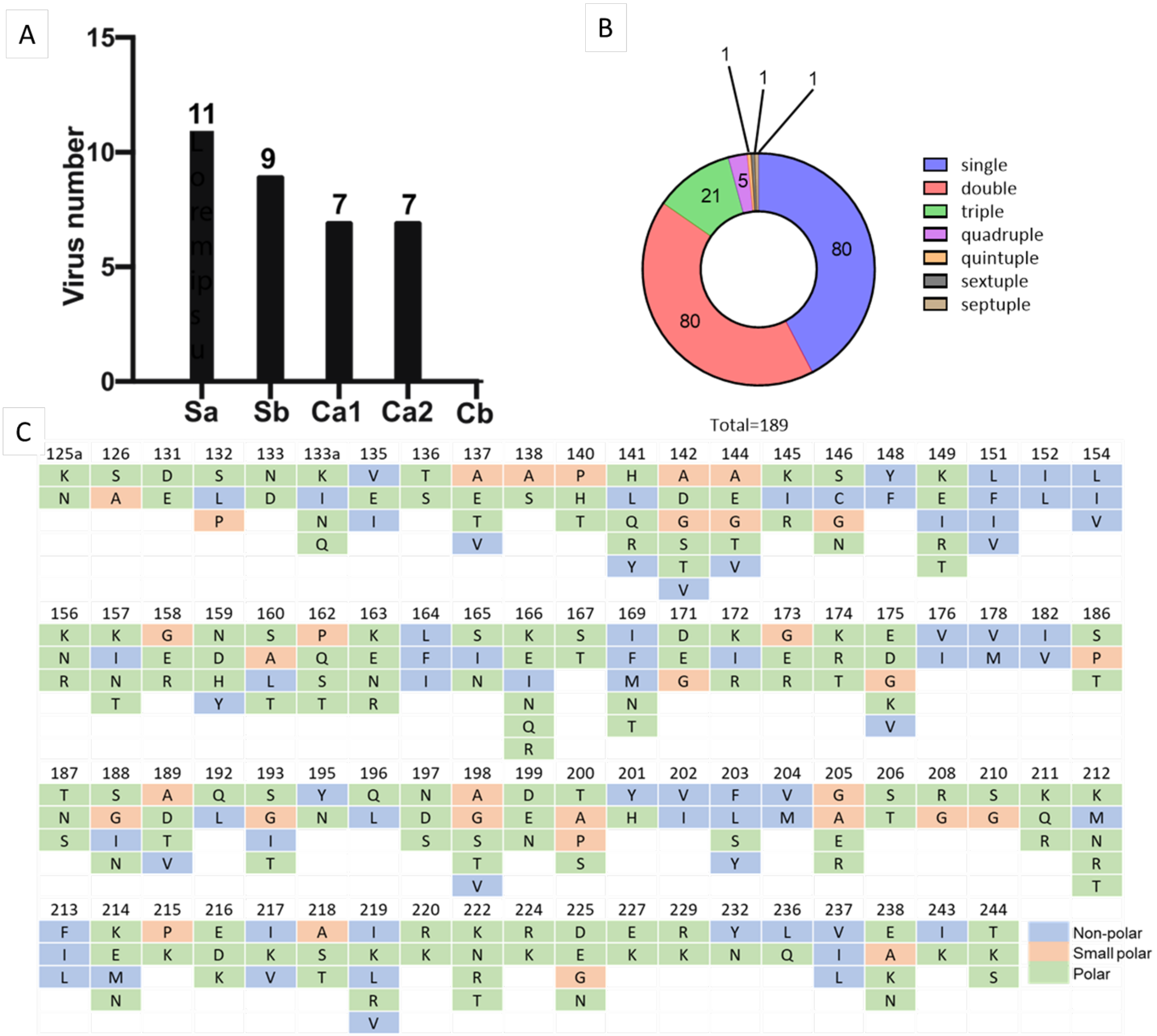

Supplementary Figure 1. Characteristics of the HA RBS mutants. A) The number of mutant viruses with at least one amino acid substitution located in the HA antibody binding sites of H1N1 viruses; B) the number of amino acid substitutions for each mutant in the HA RBS mutant library; C) biophysical properties of amino acid substitutions in the HA RBS mutants. The amino acids were grouped in three categories: nonpolar (including V, L, I, M, C, F, W, and Y) in light blue, small nonpolar (including G, A, and P) in pink, and polar/charged (including S, T, N, Q, H, D, E, K, and R) in light green. Source data are provided as a Source Data file.

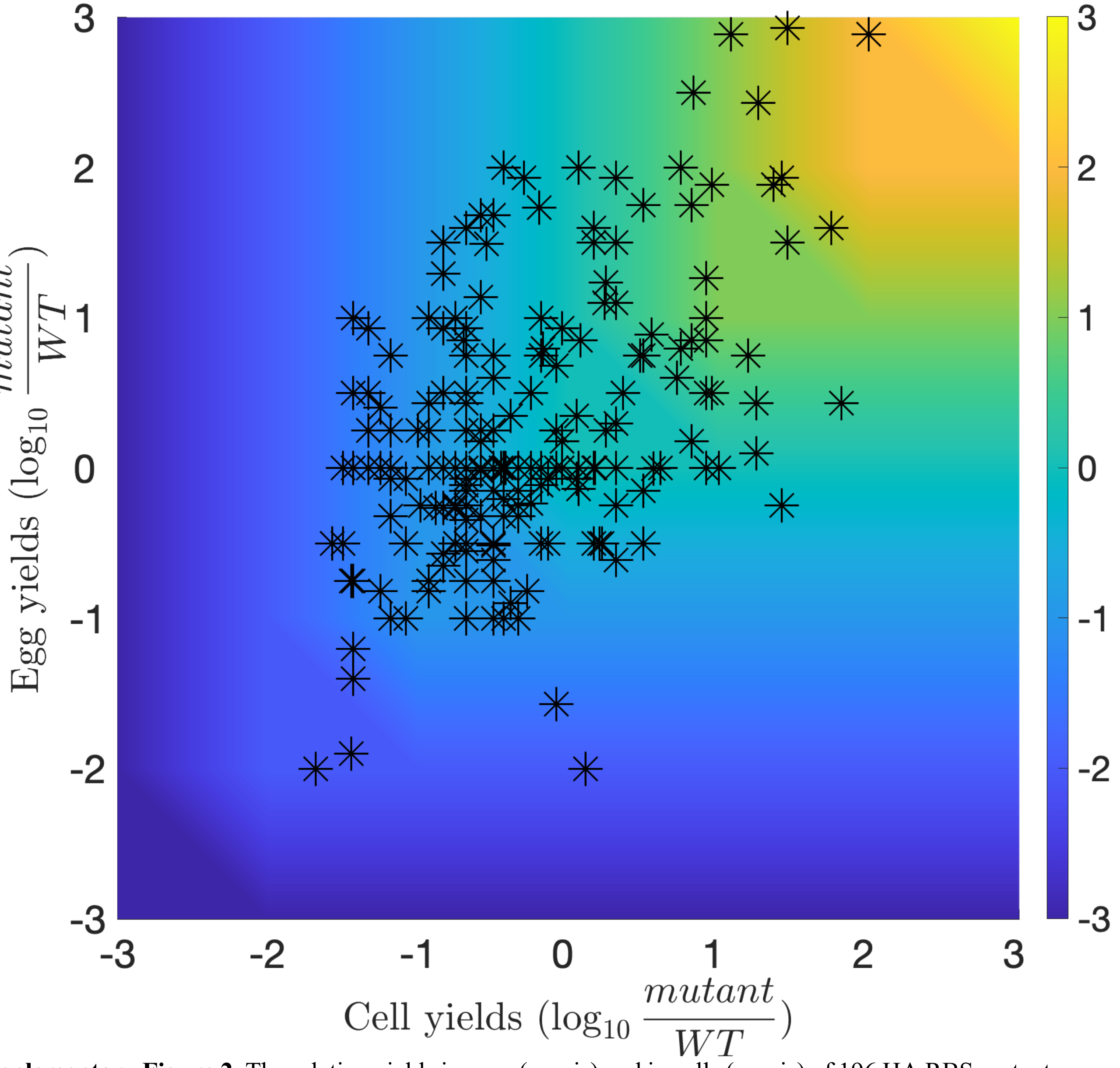

Supplementary Figure 2. The relative yields in eggs (y-axis) and in cells (x-axis) of 196 HA RBS mutants compared to the CA/04 WT virus. Source data are provided as a Source Data file.

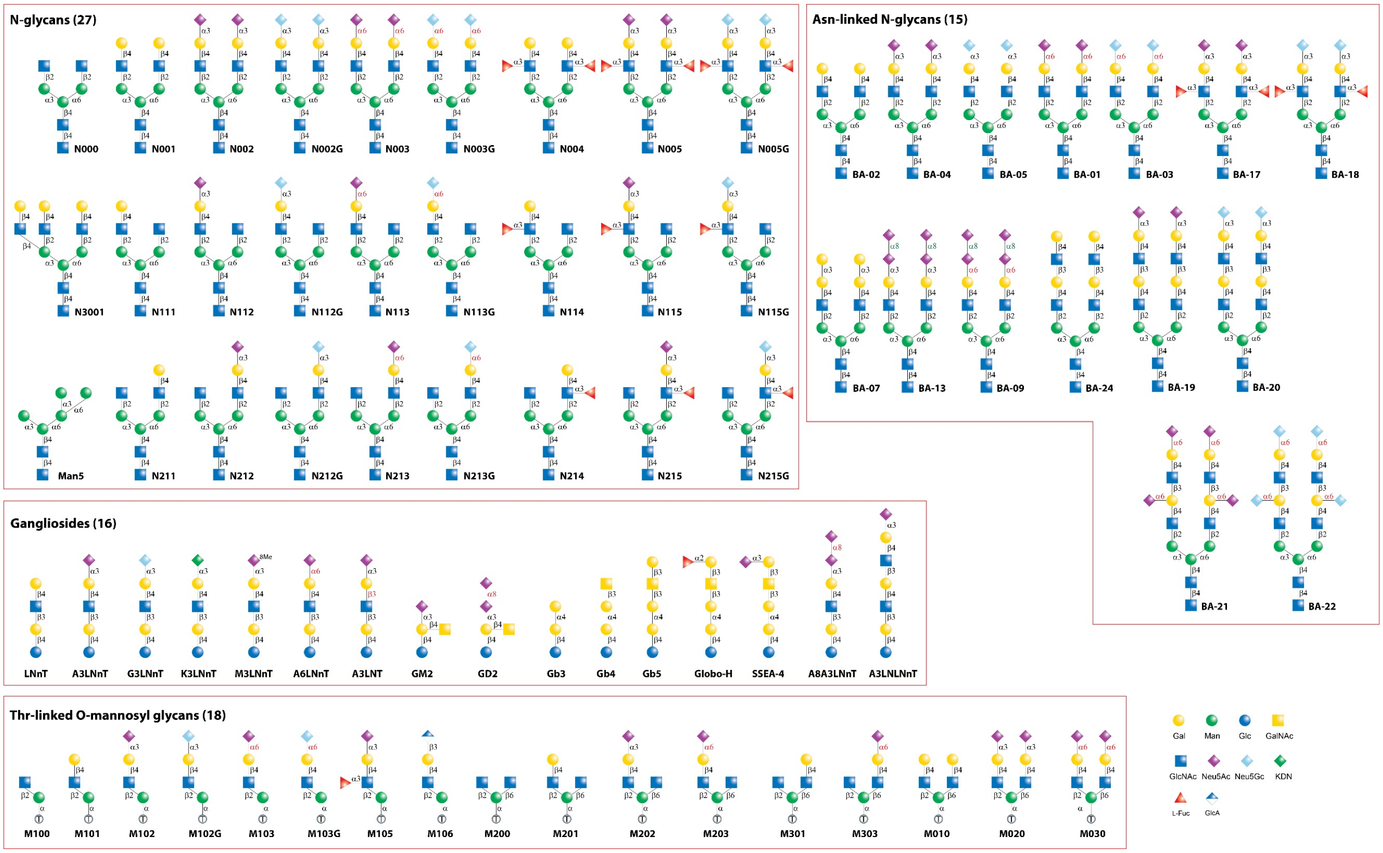

Supplementary Figure 3. List of 75 glycoforms printed on the glycan microarray.

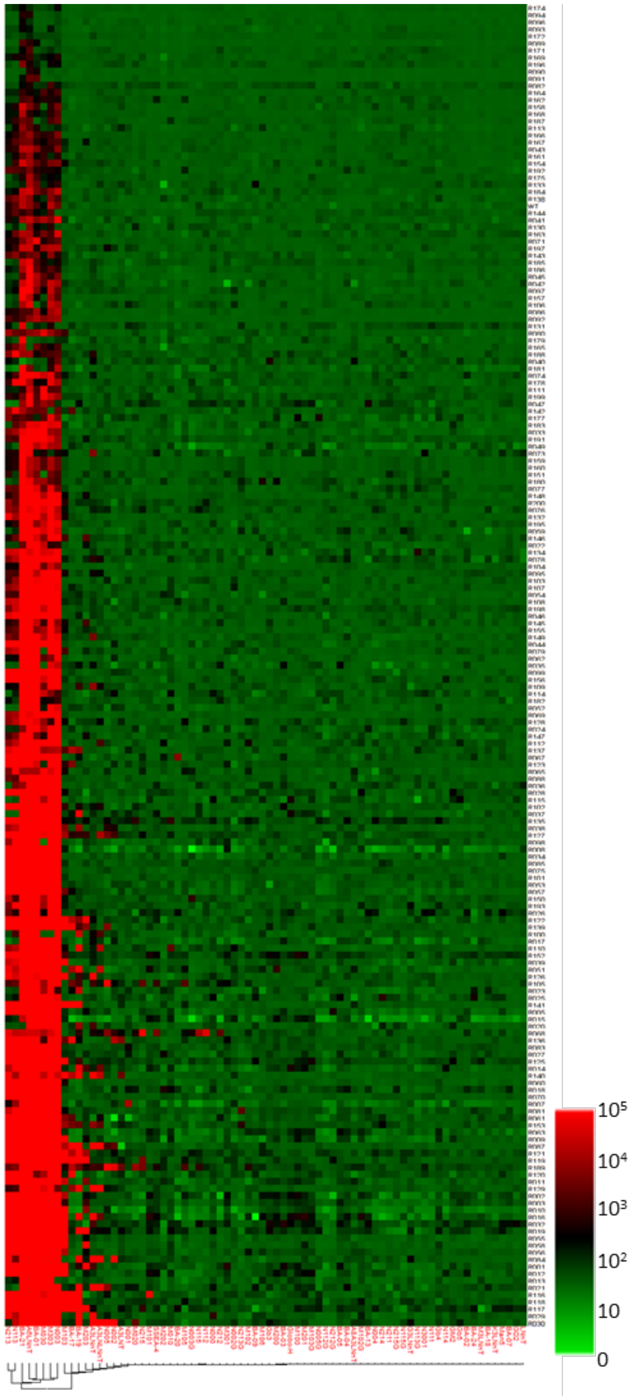

Supplementary Figure 4. Heat map illustration of binding intensity of viruses to glycans on the glycan microarray. Each row represents a HA RBS mutant and each column represents an individual glycan. The color bar represents the magnitude of the binding intensity. The linkage hierarchical clustering was performed by Hierarchical Clustering Explorer 3.0 (https://www.cs.umd.edu/hcil/multi-cluster/) to generate the heat map and tree structure. Source data are provided as a Source Data file.

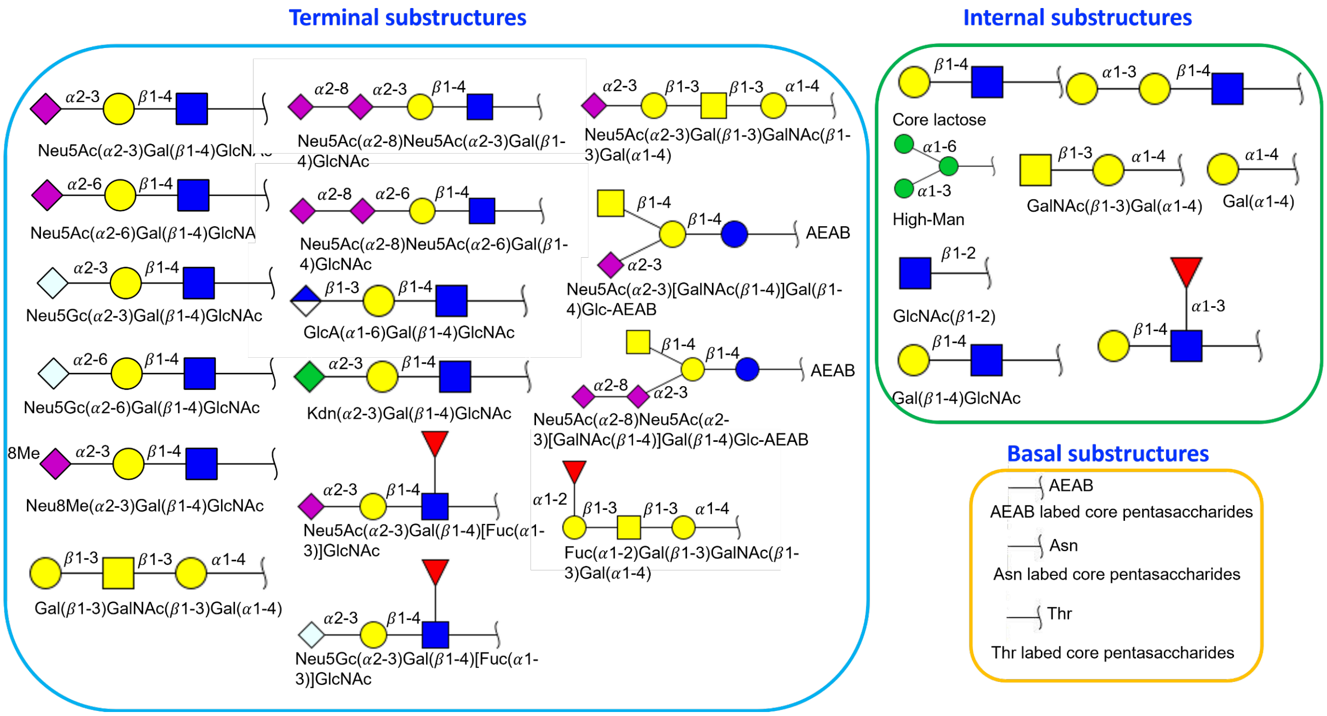

Supplementary Figure 5. List of 27 glycan substructures used in machine learning. These 27 substructures are separated into three groups: terminal, internal, or basal substructures.

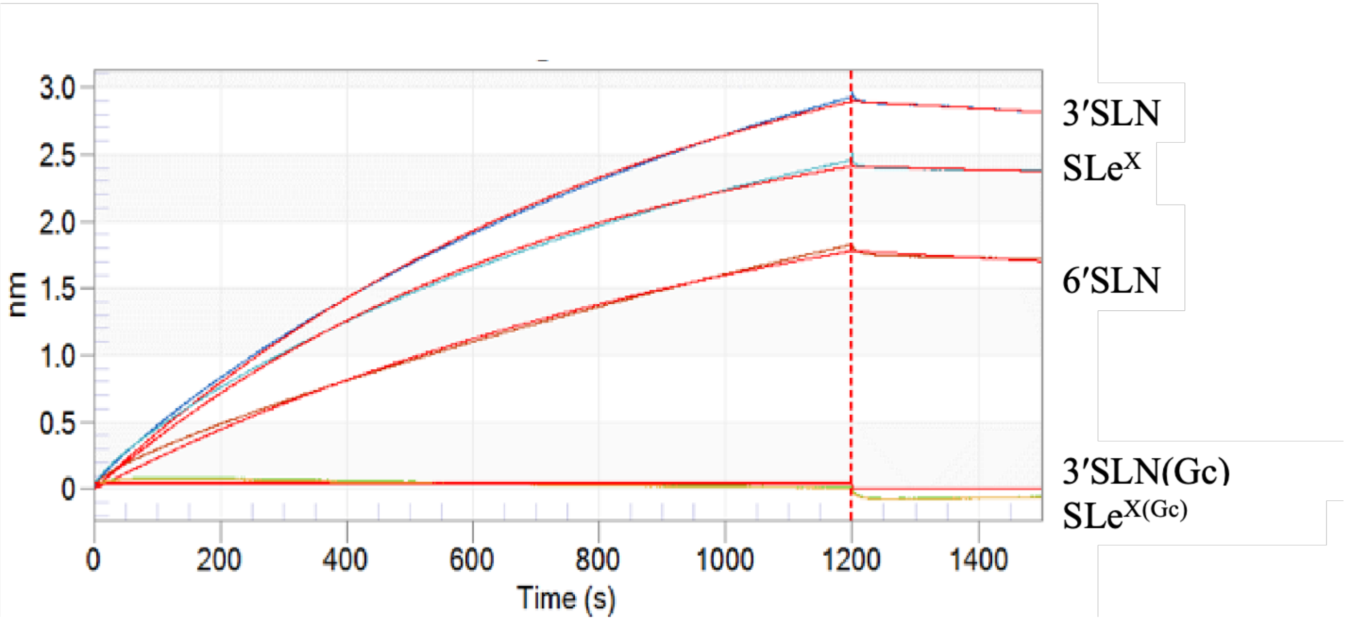

Supplementary Figure 6. Glycan binding specificity of the HA D131E-S193T-A198S mutant by Bio-Layer Interferometry analyses. Binding of viruses to five representative glycan analogs, [Neu5Acα2-3Galβ1-4GlcNAcβ (3′SLN), Neu5Acα2-6Galβ1-4GlcNAcβ (6′SLN), and Neu5Acα2-3Galβ1-4[Fucα1-3]GlcNAcβ (sLe^{X}), Neu5Gcα2-3Galβ1-4GlcNAcβ (3′SLN(Gc)), and Neu5Gcα2-3Galβ1-4[Fucα1-3]GlcNAcβ (sLe$^{X(Gc)}$)], were determined by Bio-Layer Interferometry (Pall ForteBio LLC, Fremont, CA). The streptavidin-coated biosensors were first preloaded with biotin-labeled sialic acid receptors, followed by the 1 pM each virus binding for 1,200 seconds in a standard kinetic buffer with neuraminidase inhibitors (zanamivir hydrate and oseltamivir phosphate). Sialic acid receptor concentrations were titrated to 1 μg/ml (3′SLN and 6′SLN) or 1uM (sLeX) when loading with the biotin-labeled receptors. The binding response unit (nm) was recorded at the 1,196 second time point (4 seconds before the start of dissociation).

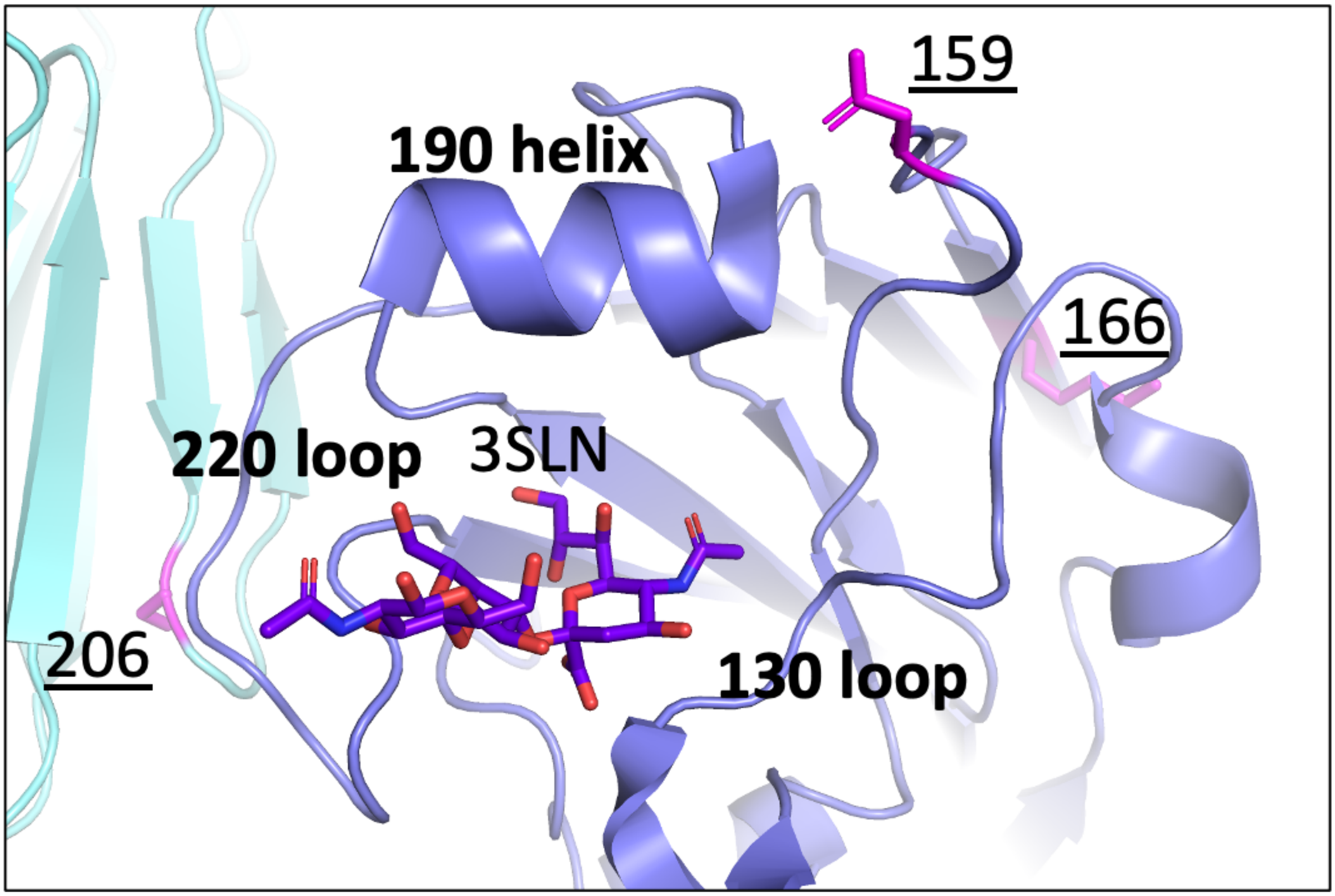

Supplementary Figure 7. Structural modeling showing the synergistic effects of amino acid substitution N159K, K166Q and S206T on the binding affinity of HA to SA2-3Gal. The results showed that residue 166 interacts with an alpha-helix at the C-terminal end of the 130-loop and the K166Q substitution may affect the conformation of the 130-loop, resulting in changes in binding affinity towards glycan receptors. In addition, residue 206 is located at the HA inter-subunit interface adjacent to the 220-loop, and the S206T substitution could potentially alter the structural conformation of the 220-loop, thus affecting the glycan receptor binding. The three-dimensional structure of HA protein was modeled based on the crystal structure of CA/04 HA in complex with 6′SLN (PDB ID# 3UBN) and 3′SLN (PDB ID# 3UBQ). Coot was first used to introduce the desired mutation to the three subunits of a HA trimer (*51*). The mutated coordinates were subsequently refined by energy minimization using Phenix (*52*). Structure figures were made using Pymol (The PyMOL Molecular Graphics System, Version 1.3, Schrödinger, LLC).

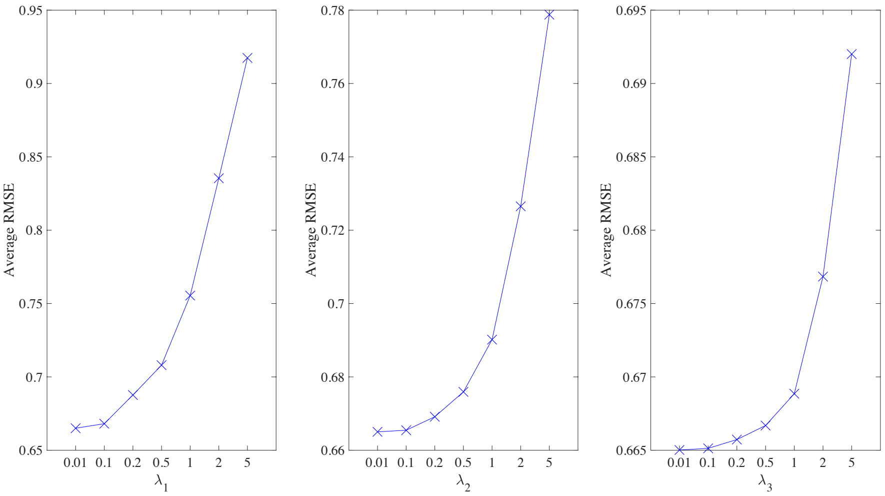

Supplementary Figure 8. Optimization of the parameters λ_1, λ_2, and $\lambda_3$used in multi-task feature learning of MTL-GGSL for antigenicity analyses. Source data are provided as a Source Data file.

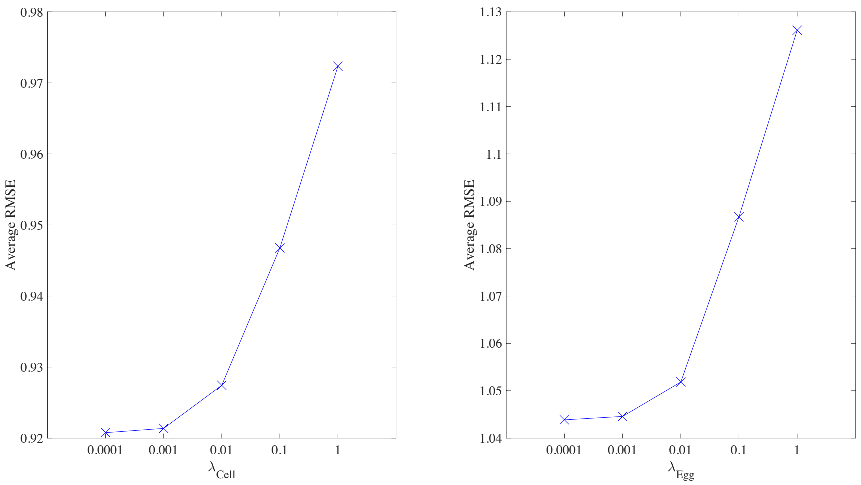

Supplementary Figure 9. Optimization of parameters λ used in GHSM for in cell and egg yield analyses. Source data are provided as a Source Data file.

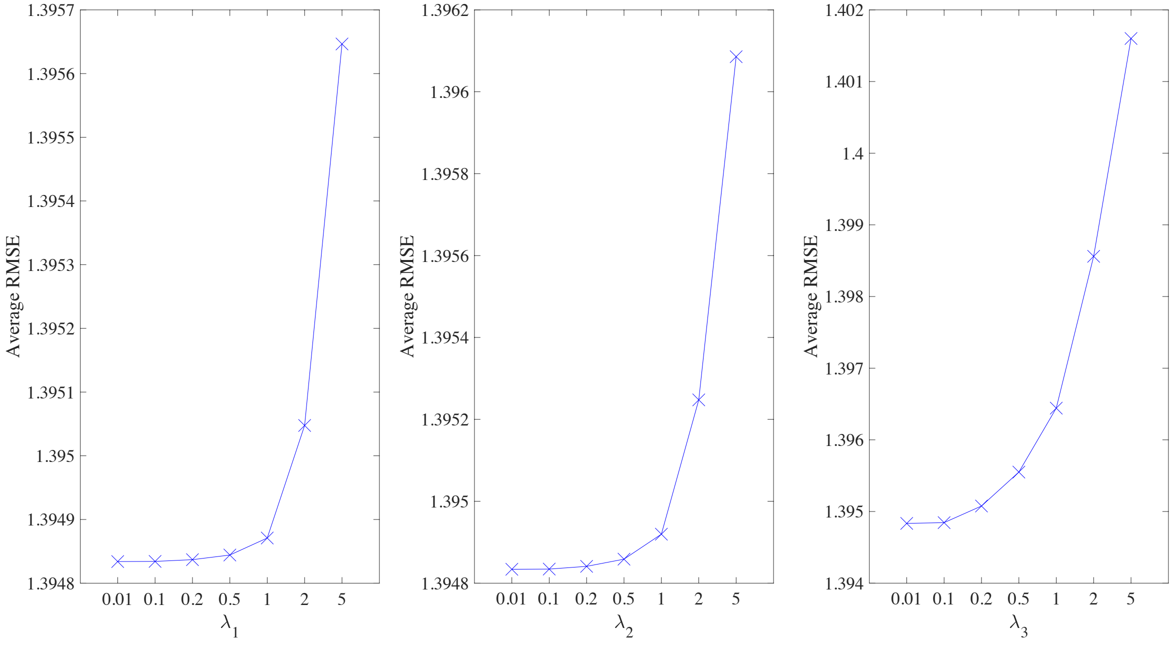

Supplementary Figure 10. Optimization of the parameters λ_1, λ_2, and λ_3 in multi-task feature learning of MTL-GGSL for glycan binding analyses in the HY^{both} group. Source data are provided as a Source Data file.

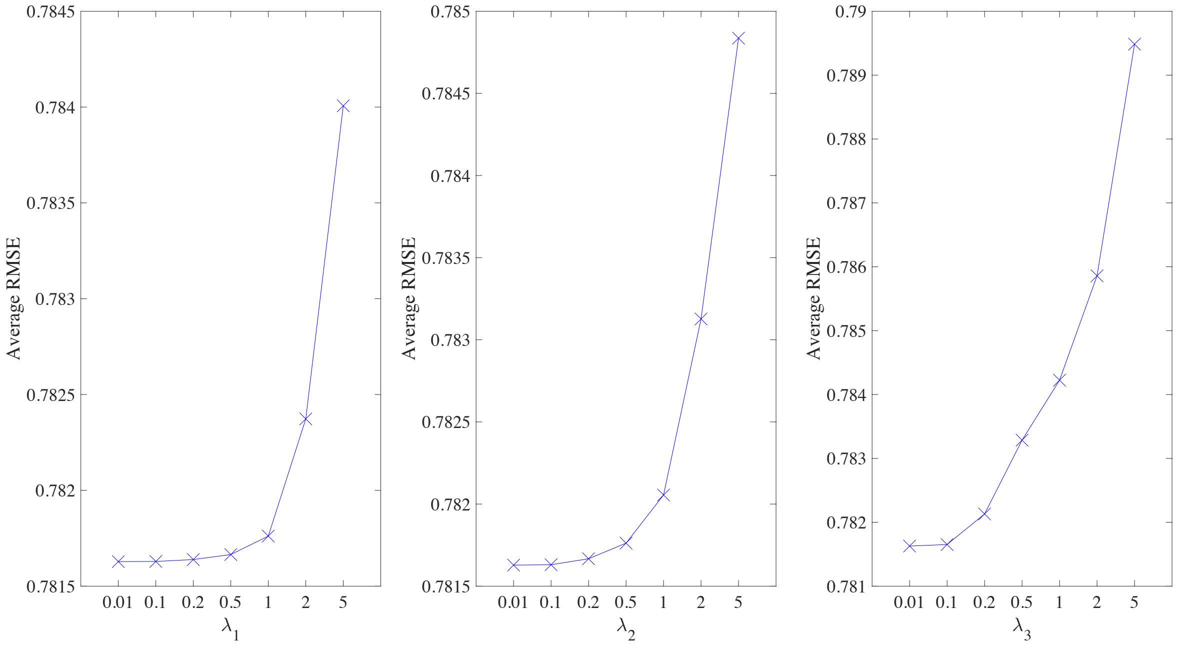

Supplementary Figure 11. Optimization of the parameters λ_1, λ_2, and λ_3 in multi-task feature learning of MTL-GGSL for glycan binding analyses in the HY^{cell} group. Source data are provided as a Source Data file.

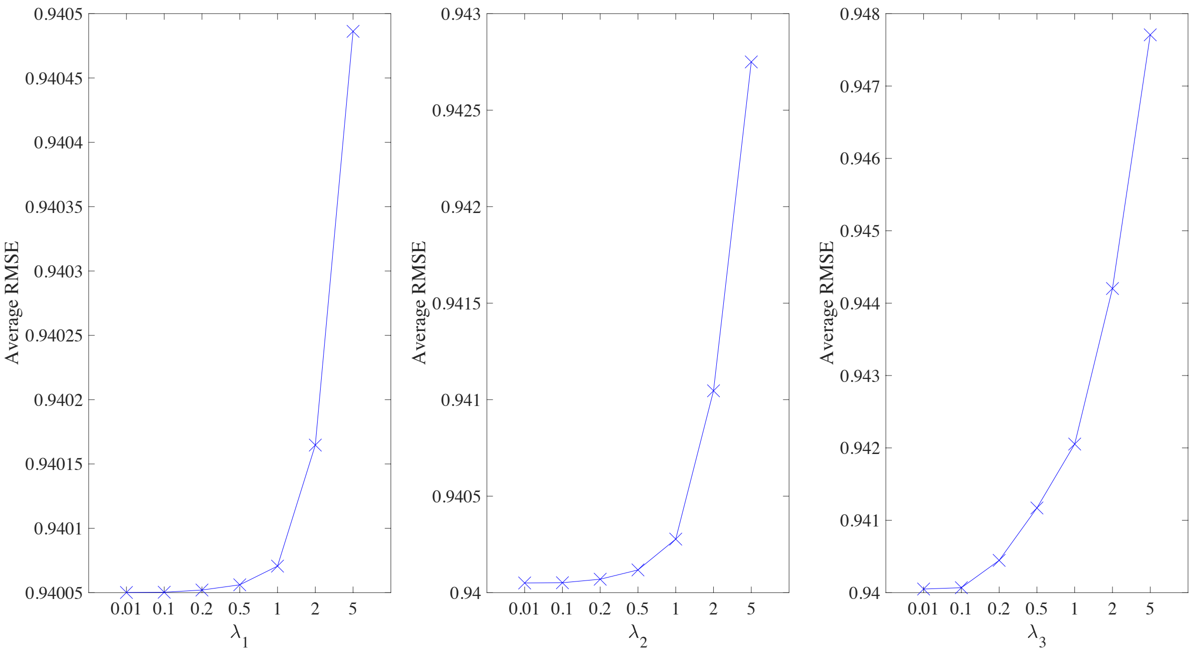

Supplementary Figure 12. Optimization of the parameters λ_1, λ_2, and λ_3 in multi-task feature learning of MTL-GGSL for glycan binding analyses in the HY^{egg} group. Source data are provided as a Source Data file.

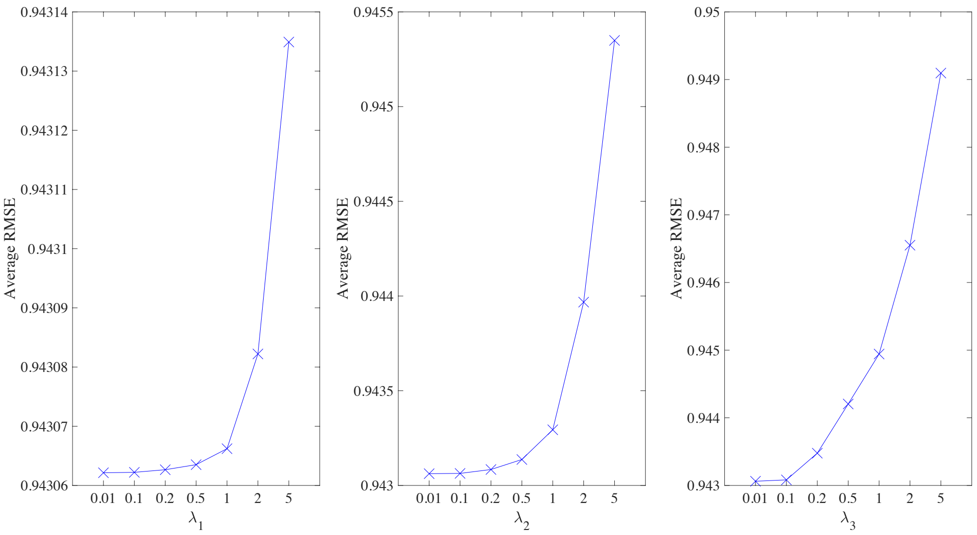

Supplementary Figure 13. Optimization of the parameters λ_1, λ_2, and λ_3 in multi-task feature learning of MTL-GGSL for glycan binding analyses in the LY^{both} group. Source data are provided as a Source Data file.

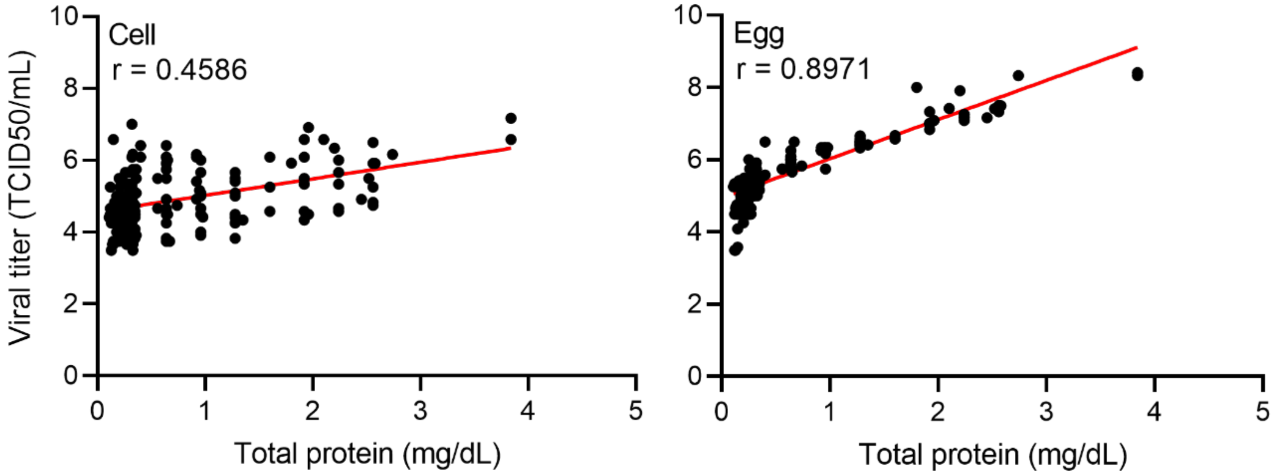

Supplementary Figure 14. Correlation between viral titration TCID50 in and the total proteins obtained from ultracentrifugation purification of supernatants from virus-infected cell or egg cultures. Pearson correlation analysis was conducted using Prism. Typically, a coefficient r between 0.4 and 0.7 is considered to indicate a moderate positive correlation, while a value above 0.7 signifies a strong positive correlation. Source data are provided as a Source Data file.

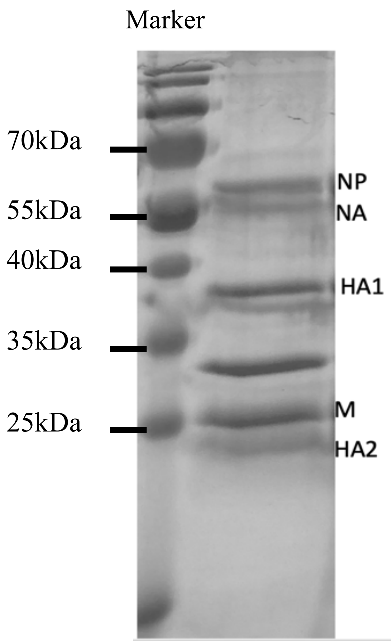

	Image J	Percentage of HA%
NP	2070.08	
NA	1679.33	
HA1	3641.33	
M	5351.54	
HA2	2428.47	
Total	15170.74	
HA: HA1+HA2	6069.79	40.01

Supplementary Figure 15. Quantification of HA proteins from egg-grown A/California/04/2009(HA, NA)×A/Puerto Rico/8/1934(H1N1). Ultracentrifugation products totaling 5 ug were treated with 500 units of PNGase F, followed by analysis using 12% SDS-PAGE. The intensities quantified by Image J are shown in the right table for five major proteins components (NP, NA, HA1, M, and HA2) identified in the SDS gel, and the HA1 and HA2 consist of 40.01% of the total proteins. Source data are provided as Supplementary Figure 16.

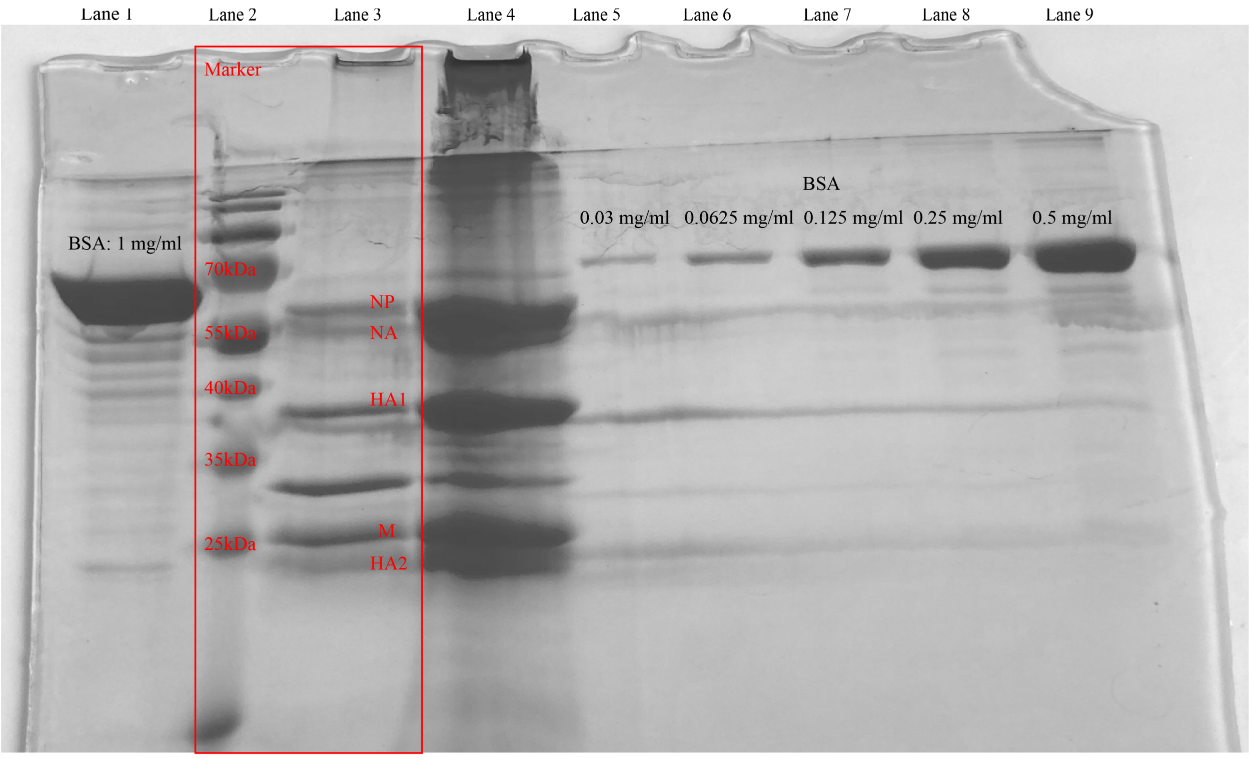

Supplementary Figure 16. Uncropped scans of blots showing the quantification of HA proteins from egg-grown A/California/04/2009(HA, NA)×A/Puerto Rico/8/1934(H1N1). The results presented in Supplementary Figure 15 are highlighted within the red box. Sample loading information for each lane is as follows: Lane 1: BSA (1 mg/ml); Lane 2: Marker; Lane 3: 5 ug virus treated with 500 units of PNGase F; Lane 4: 50 ug virus treated with 500 units of PNGase F; Lane 5: BSA (0.03 mg/ml); Lane 6: BSA (0.0625 mg/ml); Lane 7: BSA (0.125 mg/ml); Lane 8: BSA (0.25 mg/ml); Lane 9: BSA (0.5 mg/ml).

Supplementary References

1. Tibshirani, R. Regression shrinkage and selection via the LASSO. *Journal of the Royal Statistical Society (Series B)* **58**, 267-288 (1996).
2. Marquardt, D.W. & Snee, R.D.J.T.A.S. Ridge regression in practice. **29**, 3-20 (1975).
3. Friedman, J., Hastie, T. & Tibshirani, R. A note on the group lasso and a sparse group lasso. *arXiv preprint arXiv:1001.0736* (2010).
4. Zou, H. & Hastie, T. Regularization and variable selection via the elastic net. *Journal of the Royal Statistical Society (Series B)* **67**, 301-320 (2005).
5. Zhao, P., Rocha, G. & Yu, B. The composite absolute penal-ties family for grouped and hierarchical variable selection. *The Annals of Statistics* **2009**, 3468–3497 (2009).
6. Liao, Y.C., Lee, M.S., Ko, C.Y. & Hsiung, C.A. Bioinformatics models for predicting antigenic variants of influenza A/H3N2 virus. *Bioinformatics* **24**, 505-512 (2008).
7. Agor, J.K. & Ozaltin, O.Y. Models for predicting the evolution of influenza to inform vaccine strain selection. *Human vaccines & immunotherapeutics* **14**, 678-683 (2018).
8. Yao, B., Zhang, L., Liang, S. & Zhang, C. SVMTriP: a method to predict antigenic epitopes using support vector machine to integrate tri-peptide similarity and propensity. *PLoS One* **7**, e45152 (2012).
9. Du, X. et al. Mapping of H3N2 influenza antigenic evolution in China reveals a strategy for vaccine strain recommendation. *Nature communications* **3**, 709 (2012).
10. Li, H. et al. PREDAC-H5: a user-friendly tool for the automated surveillance of antigenic variants for the HPAI H5N1 virus. *Infection, genetics and evolution: journal of molecular epidemiology and evolutionary genetics in infectious diseases* **28**, 62-63 (2014).
11. Liu, M. et al. Antigenic patterns and evolution of the human influenza A (H1N1) virus. *Scientific reports* **5**, 14171 (2015).
12. Peng, Y. et al. A universal computational model for predicting antigenic variants of influenza A virus based on conserved antigenic structures. *Scientific reports* **7**, 42051 (2017).
13. Zhou, X., Yin, R., Kwoh, C.-K. & Zheng, J. A context-free encoding scheme of protein sequences for predicting antigenicity of diverse influenza A viruses. *BMC genomics* **19**, 145-154 (2018).
14. Zhou, X., Yin, R., Kwoh, C.K. & Zheng, J. A context-free encoding scheme of protein sequences for predicting antigenicity of diverse influenza A viruses. *BMC Genomics* **19**, 936 (2018).
15. Yao, Y. et al. Predicting influenza antigenicity from Hemagglutintin sequence data based on a joint random forest method. *Scientific reports* **7**, 1545 (2017).
16. Zeller, M.A. et al. Machine Learning Prediction and Experimental Validation of Antigenic Drift in H3 Influenza A Viruses in Swine. *mSphere* **6** (2021).
17. Waters, K. et al. Triple reassortment increases compatibility among viral ribonucleoprotein genes of contemporary avian and human influenza A viruses. *PLoS Pathog* **17**, e1009962 (2021).
18. Cai, Z. et al. Identifying antigenicity-associated sites in highly pathogenic H5N1 influenza virus hemagglutinin by using sparse learning. **422**, 145-155 (2012).
19. Sun, H. et al. Inferring influenza virus antigenicity using sequence data. **4**, 4 (2013).
20. Yang, J., Zhang, T. & Wan, X.-F.J.P.o. Sequence-based antigenic change prediction by a sparse learning method incorporating co-evolutionary information. **9**, e106660 (2014).

21. Harvey, W.T. et al. Identification of low-and high-impact hemagglutinin amino acid substitutions that drive antigenic drift of influenza A (H1N1) viruses. **12**, e1005526 (2016).
22. Lee, M.-S. & Chen, J.S.-E. Predicting antigenic variants of influenza A/H3N2 viruses. *Emerging infectious diseases* **10**, 1385 (2004).
23. Neher, R.A., Bedford, T., Daniels, R.S., Russell, C.A. & Shraiman, B.I. Prediction, dynamics, and visualization of antigenic phenotypes of seasonal influenza viruses. *Proceedings of the National Academy of Sciences* **113**, E1701-E1709 (2016).
24. Bedford, T. et al. Integrating influenza antigenic dynamics with molecular evolution. *elife* **3**, e01914 (2014).
25. Cui, H. et al. Using multiple linear regression and physicochemical changes of amino acid mutations to predict antigenic variants of influenza A/H3N2 viruses. *Bio-medical materials and engineering* **24**, 3729-3735 (2014).
26. Rahman, T., Mahapatra, M., Laing, E. & Jin, Y. Evolutionary non-linear modelling for selecting vaccines against antigenically variable viruses. *Bioinformatics* **31**, 834-840 (2015).
27. Qiu, J., Qiu, T., Yang, Y., Wu, D. & Cao, Z. Incorporating structure context of HA protein to improve antigenicity calculation for influenza virus A/H3N2. *Scientific reports* **6**, 1-9 (2016).
28. Liao, Y.-C., Lee, M.-S., Ko, C.-Y. & Hsiung, C.A. Bioinformatics models for predicting antigenic variants of influenza A/H3N2 virus. *Bioinformatics* **24**, 505-512 (2008).
29. Huang, J.-W., King, C.-C. & Yang, J.-M. Co-evolution positions and rules for antigenic variants of human influenza A/H3N2 viruses. *BMC bioinformatics* **10**, 1-10 (2009).
30. Abbas, M.E., Chengzhang, Z., Fathalla, A. & Xiao, Y. End-to-end antigenic variant generation for H1N1 influenza HA protein using sequence to sequence models. *Plos One* **17**, e0266198 (2022).
31. Yin, R., Thwin, N.N., Zhuang, P., Lin, Z. & Kwoh, C.K. IAV-CNN: a 2D convolutional neural network model to predict antigenic variants of influenza A virus. *IEEE/ACM Transactions on Computational Biology and Bioinformatics* **19**, 3497-3506 (2021).
32. Li, L. et al. Multi-task learning sparse group lasso: a method for quantifying antigenicity of influenza A (H1N1) virus using mutations and variations in glycosylation of Hemagglutinin. **21**, 1-22 (2020).
33. Han, L., Zhang, Y., Wan, X.-F. & Zhang, T. in Proceedings of the 22nd ACM SIGKDD International Conference on Knowledge Discovery and Data Mining 865-874 (2016).
34. Han, L. et al. Graph-guided multi-task sparse learning model: a method for identifying antigenic variants of influenza A (H3N2) virus. **35**, 77-87 (2019).
35. Han, W. et al. Predicting the antigenic evolution of SARS-COV-2 with deep learning. *Nat Commun* **14**, 3478 (2023).
36. Yang, L. et al. Mutations associated with egg adaptation of influenza A (H1N1) pdm09 virus in laboratory based surveillance in China, 2009–2016. **1**, 41-45 (2019).
