# Supplementary material for: MAIVeSS: streamlined selection of antigenically matched, high-yield viruses for seasonal influenza vaccine production
Source: Nat Commun. 2024 Feb 6;15:1128. doi: 10.1038/s41467-024-45145-x (PMC10847134; doi:10.1038/s41467-024-45145-x)
Supplement: Supplementary file 3 — Description of Additional Supplementary Files [file 41467_2024_45145_MOESM3_ESM.pdf]

## Description of Additional Supplementary Files:

**Supplementary Data 1:** The performance comparison between MAIVeSS and the other machine learning models for antigenic analyses.

**Supplementary Data 2:** The performance comparison between MAIVeSS and the other machine learning models for yield analyses.

**Supplementary Data 3:** The performance comparison between MAIVeSS and the other machine learning models for glycan binding analyses.

**Supplementary Data 4:** List of the wild type CA/04 and the HA RBS mutant viruses and their associated antigenicity, virus yield in egg and cells, and glycan binding properties. Of note, some mutants lost the ability to hemagglutinate turkey RBCs, precluding hemagglutination inhibition analyses.

**Supplementary Data 5:** Absolute local weights ($w^{local}$) for residues associated with antigenicity of A(H1N1)pdm09 viruses determined by MAIVeSS.

**Supplementary Data 6:** Amino acid substitutions associated with glycan binding properties selected by machine learning for 2009 H1N1 viruses.

**Supplementary Data 7:** Global weights for glycan binding-related features determined by MAIVeSS.

**Supplementary Data 8:** Absolute local weights for glycan binding-related features associated with $HY^{both}$ viruses determined by MAIVeSS.

**Supplementary Data 9:** Absolute local weights for glycan binding-related features associated with $HY^{cell}$ viruses determined by MAIVeSS.

**Supplementary Data 10:** Absolute local weights for glycan binding-related features associated with $HY^{egg}$ viruses determined by MAIVeSS.

**Supplementary Data 11:** Absolute local weights for glycan binding-related features associated with $LY^{both}$ viruses determined by MAIVeSS.

**Supplementary Data 12:** The antigenic and growth properties of 11,424 epidemic strains (2009-2020) predicted by MAIVeSS.

**Supplementary Data 13:** Residues associated with high yield virus strains (2009-2020) predicted by MAIVeSS.

**Supplementary Data 14:** The surface sites calculated from GetArea (http://curie.utmb.edu/getarea.html) using HA protein of pdmH1N1 virus as the template (PDB ID 3LZG [https://doi.org/10.2210/pdb3LZG/pdb])).

**Supplementary Data 15:** The hemagglutination inhibition (HAI) titers used for the model developments, including seasonal H1N1 viruses (subtables 1-3), swine influenza virus (subtables 4 and 5), and 2009 H1N1 viruses (subtable 6). The value of 0 means "missing data", and the value "<10" denotes a low reactor in an HAI assay.
