# Supplementary material for: MAIVeSS: streamlined selection of antigenically matched, high-yield viruses for seasonal influenza vaccine production
Source: Nat Commun. 2024 Feb 6;15:1128. doi: 10.1038/s41467-024-45145-x (PMC10847134; doi:10.1038/s41467-024-45145-x)
Supplement: Supplementary file 19 — Reporting Summary [file 41467_2024_45145_MOESM19_ESM.pdf]

nature portfolio

Corresponding author(s): XIU-FENG WAN

Last updated by author(s): Dec 12, 2023

Reporting Summary

Nature Portfolio wishes to improve the reproducibility of the work that we publish. This form provides structure for consistency and transparency in reporting. For further information on Nature Portfolio policies, see our Editorial Policies and the Editorial Policy Checklist.

Statistics

For all statistical analyses, confirm that the following items are present in the figure legend, table legend, main text, or Methods section.

n/a	Confirmed	
[ ]	[x]	The exact sample size (*n*) for each experimental group/condition, given as a discrete number and unit of measurement
[ ]	[x]	A statement on whether measurements were taken from distinct samples or whether the same sample was measured repeatedly
[x]	[ ]	The statistical test(s) used AND whether they are one- or two-sided<br>*Only common tests should be described solely by name; describe more complex techniques in the Methods section.*
[x]	[ ]	A description of all covariates tested
[x]	[ ]	A description of any assumptions or corrections, such as tests of normality and adjustment for multiple comparisons
[x]	[ ]	A full description of the statistical parameters including central tendency (e.g. means) or other basic estimates (e.g. regression coefficient) AND variation (e.g. standard deviation) or associated estimates of uncertainty (e.g. confidence intervals)
[x]	[ ]	For null hypothesis testing, the test statistic (e.g. *F*, *t*, *r*) with confidence intervals, effect sizes, degrees of freedom and *P* value noted<br>*Give P values as exact values whenever suitable.*
[x]	[ ]	For Bayesian analysis, information on the choice of priors and Markov chain Monte Carlo settings
[ ]	[x]	For hierarchical and complex designs, identification of the appropriate level for tests and full reporting of outcomes
[ ]	[x]	Estimates of effect sizes (e.g. Cohen's *d*, Pearson's *r*), indicating how they were calculated

Our web collection on statistics for biologists contains articles on many of the points above.

Software and code

Policy information about availability of computer code

Data collection	MEGA (Molecular Evolutionary Genetics Analysis, Version 10.1.8) was utilized to conduct the multiple sequence alignment, enabling the identification of specific amino acids across protein sequences.
Data analysis	The sparse learning models developed in this study were implemented and validated using Matlab (Version R2019a) on a Windows system. GETAREA software (http://curie.utmb.edu/getarea.html) was used to predict whether these residues were on HA's surface. Virus receptor binding data derived from BLI assays with an Octet RED instrument were analyzed using GraphPad Prism 8 (https://www.graphpad.com/scientific-software/prism/). For introducing desired mutations to the three subunits of a HA trimer, Coot (https://www2.mrc-lmb.cam.ac.uk/personal/pemsley/coot/) was employed. Energy minimization in structural modeling was performed using Phenix (https://phenix-online.org). Structure figures were generated using Pymol (The PyMOL Molecular Graphics System, Version 1.3, Schrödinger, LLC). Bar graphs were created using Microsoft Excel version 16.74. Finally, the antigenic cartography was generated using AntigenMap (https://sysbio-lab.mu.hekademeia.org/AntigenMap). The source codes for the model development in this study can be accessed through Github at https://github.com/FluSysBio/MAIVeSS and also through Code Ocean at https://doi.org/10.24433/CO.8910619.v1. The MAIVeSS webserver can be accessed at http://sysbio.missouri.edu/software/MAIVeSS.

For manuscripts utilizing custom algorithms or software that are central to the research but not yet described in published literature, software must be made available to editors and reviewers. We strongly encourage code deposition in a community repository (e.g. GitHub). See the Nature Portfolio guidelines for submitting code & software for further information.

Data

Policy information about availability of data

All manuscripts must include a data availability statement. This statement should provide the following information, where applicable:

- Accession codes, unique identifiers, or web links for publicly available datasets
- A description of any restrictions on data availability
- For clinical datasets or third party data, please ensure that the statement adheres to our policy

The serological data for the vaccinate candidates generated in this study are available in Table 3. The list of the wild type CA/04 and the HA RBS mutant viruses and their associated antigenicity, virus yield in egg and cells, and glycan binding properties generated in this study are available at Supplementary Data 4. The serological data used in model development were from public sources and included HAI titers generated between 1,015 viruses and 194 serum samples (Supplementary Data 15). A total of 11,424 A(H1N1)pdm09 HA protein sequences from 2009 to 2020 were obtained from GISAID (https://gisaid.org). The glycans printed on microarray array are available in Supplementary Figure 1. The GISAID accession numbers for the epidemic A(H1N1)pdm09 strains are available from Supplementary Data 12. To access the GISAID database (https://gisaid.org), users need to log in following the instructions provided by the GISAID database. Once logged in, the GISAID database enables users to search and retrieve sequence and metadata data using either a specific accession number or a specific strain name. The three-dimensional structure of the HA protein was modeled by referencing the crystal structures of CA/04 HA (PDB ID# 3LZG) [https://doi.org/10.2210/pdb3LZG/pdb] in complex with 6'SLN (PDB ID# 3UBN) [https://doi.org/10.2210/pdb3UBN/pdb] and 3 SLN (PDB ID# 3UBQ) [https://doi.org/10.2210/pdb3UBQ/pdb]. Additionally, the original data utilized for generating bar graphs and geospatial visualizations can be accessed in the Source Data file. Source data are provided with this paper.

Research involving human participants, their data, or biological material

Policy information about studies with human participants or human data. See also policy information about sex, gender (identity/presentation), and sexual orientation and race, ethnicity and racism.

Reporting on sex and gender	Not applicable
Reporting on race, ethnicity, or other socially relevant groupings	Not applicable
Population characteristics	Not applicable
Recruitment	Not applicable
Ethics oversight	Not applicable

Note that full information on the approval of the study protocol must also be provided in the manuscript.

Field-specific reporting

Please select the one below that is the best fit for your research. If you are not sure, read the appropriate sections before making your selection.

☐ Life sciences ☐ Behavioural & social sciences ☒ Ecological, evolutionary & environmental sciences

For a reference copy of the document with all sections, see nature.com/documents/nr-reporting-summary-flat.pdf

Ecological, evolutionary & environmental sciences study design

All studies must disclose on these points even when the disclosure is negative.

Study description	In this study, we retrieved HA sequences from public databases, encompassing a total of 11,424 2009 H1N1 viruses. Subsequently, we employed a Machine-learning Assisted Influenza Vaccine Strain Selection framework (MAIVeSS) to quantify their replication yields in both eggs and cells. We proceeded to examine the temporal distribution of these high-yield influenza viruses within human populations, as they hold potential for direct utilization as vaccine seed strains. Notably, no additional treatment factors, interactions, or design structures were introduced in this study. This study represents a single experiment as it represents the application of the model.
Research sample	We retrieved HA sequences from a comprehensive dataset comprising 11,424 2009 H1N1 viruses, all of which were sourced from the GISAID database (https://gisaid.org). These sequences were specifically selected to represent the 2009 H1N1 seasonal influenza A viruses responsible for human infections during seasonal influenza outbreaks, spanning from 2009 to 2020.
Sampling strategy	No data were omitted or excluded from our analyses.
Data collection	A) The H1N1 HA sequence data utilized in our evolutionary and ecological study, through model application, were downloaded from public databases by Cheng Gao.<br><br>B) Additional data collection for machine learning model development are shown as below:<br><br>1) Serological data: Hemagglutination and HAI assays were performed by using 0.5% turkey erythrocytes as described by the WHO

Global Influenza Surveillance Network Manual for the Laboratory Diagnosis and Virological Surveillance of Influenza. Turkey erythrocytes were obtained from Lampire Biological Products (Everett, PA). The turkey erythrocytes were washed three times with 1 × PBS (pH 7.2) before use and then diluted to 0.5% in 1 × PBS (pH 7.2). Feng Wen recorded the data.

2) Yield data: To evaluate the effect of mutations on viral yield, we performed cell culture assays and embryonated egg assays. For the cell culture assays, we inoculated MDCK cells with each influenza virus at a multiplicity of infection of 0.001 and incubated the cells at 37°C with 5% CO2 for 1 hour. After incubation, the inocula were removed, and the cells were washed twice with phosphate-buffered saline (PBS). Then, the cells were incubated with Opti-MEM I (GIBCO, Grand Island, NY) containing TPCK-trypsin (1 µg/ml) at 37°C with 5% CO2. After 48 hours, 200 µl of supernatants were collected, aliquoted, and stored at -80°C until use. For the embryonated egg assays, 9-day-old specific pathogen-free chicken eggs were inoculated with 200 TCID50 of each virus and incubated at 37°C for 72 hours, and allantoic fluid were collected. The viral titers in the samples from both the MDCK cells and the embryonated eggs were determined using TCID50 assays in MDCK cells. Feng Wen and Minhui Guan recorded the data.

3) Glycan microarray data: To identify unique substructures bound specific sets of mutants, a glycan microarray with 75 glycoforms were printed on N-hydroxysuccinimide (NHS)–derivatized slides as described previously.23 The 75 glycans were selected to represent four different glycan categories, including N-glycans, Asn-linked N-glycans, Gangliosides, Thr-linked O-mannosyl glycans (SI Fig. S1). These glycans on the microarray have the same base structures and spacer arms but different terminal structures. The glycans were printed in replicates of four in a subarray, and sixteen subarrays were printed on each glass slide. All glycans were prepared at a concentration of 100 mM in phosphate buffer (100 mM sodium phosphate buffer, pH 8.5). The slides were fitted with a 16-chamber adapter to separate the subarrays into individual wells for assay. The unreacted NHS groups on the slides are blocked with 50 mM ethanolamine in 50 mM sodium borate buffer (pH 9.2) at 4 °C for 1 hour and then the slides are rinsed with water. Before the assay, slides were rehydrated for 5 min in TSMW buffer (20 mM Tris-HCl, 150 mM NaCl, 0.2 mM CaCl2, and 0.2 mM MgCl2, 0.05% Tween). Viruses are purified by sucrose density gradient ultracentrifugation and titrated to about 32, 000 hemagglutination units/ml. Then 10 µl of 1.0 M sodium bicarbonate (pH 9.0) was added to 80 µl of virus, and the virus was incubated with 10 µg of Alexa Fluor 488 NHS Ester (Succinimidyl Ester; Invitrogen, Carlsbad, CA) for 1 h at 25°C. After overnight dialysis to remove excess Alexa 488, viruses HA titer were checked and then bound to glycan array. Labeled viruses were incubated on the slide at 4°C for 2 h, washed, and centrifuged briefly before being scanned with an InnoScan 1100 AL fluorescence imager (Innopsys, Carbonne, France). Feng Wen recorded the data.

4) Biolayer interferometry assays (BLI) data: The virus receptor binding affinities were determined by BLI with an Octet RED instrument (Pall ForteBio, Menlo Park, CA). Five biotinylated glycan analogs, Neu5Acα2-3Galβ1-4GlcNacβ-PAA-biotin (3′SLN), Neu5Acα2-6Galβ1-4GlcNacβ-PAA-biotin (6′SLN) (Lectinity Holdings, Moscow, Russia), Neu5Acα2-3Galβ1-4(Fucβ1-3)GlcNacβ-PAA-biotin (sLeX), Neu5Gcα2-3Galβ1-4GlcNAcβ-PAA-biotin (3′SLN(Gc)), or Neu5Gcα2-3Galβ1-4(Fucβ1-3]GlcNAcβ-PAA-biotin (SLeX(Gc))] were used. Among them, SLeX, 3′SLN(Gc), and SLeX(Gc) were synthesized. The glycans were preloaded onto streptavidin-coated biosensors at up to 0.3 µg/ml for 5 minutes in 1 × kinetic buffer (Pall ForteBio, Menlo Park, CA). Each test virus was diluted to a final concentration of 100 pM with 1 × kinetic buffer containing 10 µM oseltamivir carboxylate (American Radiolabeled Chemicals, St. Louis, MO) and zanamivir (Sigma-Aldrich, St. Louis, MO) to prevent cleavage of the receptor analogs by NA proteins of virus. Association was measured for 30 minutes at 25°C. Beatriz Praena recorded the data.

5) Sequence and serological data from public sources used in training: The sequences and HAI data for other sources were collected from CDC Influenza vaccines report (2009-2016); Shu B, Garten R, Emery S, Balish A, Cooper L, Sessions W, Deyde V, Smith C, Berman L, Klimov A, Lindstrom S, Xu X. Genetic analysis and antigenic characterization of swine origin influenza viruses isolated from humans in the United States, 1990-2010. Virology. 2012 Jan 5;422(1):151-60. doi: 10.1016/j.virol.2011.10.016. Epub 2011 Nov 10. PMID: 22078166; Bedford T, Suchard MA, Lemey P, Dudas G, Gregory V, Hay AJ, McCauley JW, Russell CA, Smith DJ, Rambaut A. Integrating influenza antigenic dynamics with molecular evolution. Elife. 2014;3:e01914. doi: 10.7554/eLife.01914. Epub 2014 Feb 4. PMID: 24497547; PMCID: PMC3909918; Lorusso A, Vincent AL, Harland ML, Alt D, Bayles DO, Swenson SL, Gramer MR, Russell CA, Smith DJ, Lager KM, Lewis NS. Genetic and antigenic characterization of H1 influenza viruses from United States swine from 2008. J Gen Virol. 2011 Apr;92(Pt 4):919-30. doi: 10.1099/vir.0.027557-0. Epub 2010 Dec 22. PMID: 21177926; PMCID: PMC3133703. Cheng Gao recorded the data.

6) Structural data from public sources: The crystal structure of CA/04 HA in complex with 6′SLN (PDB ID# 3UBN) and 3′SLN (PDB ID# 3UBQ) were obtained from Protein Data Bank (https://www.rcsb.org). Jieze Zhang recorded the data.

Timing and spatial scale

The A(H1N1)pdm09 HA sequences used in evolutionary and ecology study represent all possible A(H1N1)pdm09 epidemic data spanning from 2009 to 2020 and from worldwide.
The serological data employed in the development of our model encompass a wide range of years. Specifically, for seasonal H1N1 viruses, the data span from 1977 to 2009, while for A(H1N1)pdm09 viruses, the data cover the period from 2009 to 2016. It's noteworthy that these serological data were collected from a global perspective. Additionally, the serological data for subtype H1 swine influenza viruses were obtained from the years 1930 through 2008, and the source for this data was exclusively the United States.

Data exclusions

All available data were included in the analyses without any exclusions.

Reproducibility

For the purpose of reproducibility, we have uploaded our data and code to GitHub at the following link: [https://github.com/FluSysBio/MAIVeSS].

Randomization

To validate the model, the bootstrap was performed by performing 100 independent experiments. During each, 80% of the training data were randomly selected, and the randomization were generated using the rand() implemented in the Matlab.

Blinding

The Investigators were not blinded to allocation during experiments and outcome assessment. When selecting samples for validation, it is essential for the investigators to identify the strains that are expected to yield high results. To mitigate the risk of potential biases, we have incorporated mock controls into each experiment.

Did the study involve field work? ☐ Yes ☒ No

Reporting for specific materials, systems and methods

We require information from authors about some types of materials, experimental systems and methods used in many studies. Here, indicate whether each material, system or method listed is relevant to your study. If you are not sure if a list item applies to your research, read the appropriate section before selecting a response.

Materials & experimental systems

n/a	Involved in the study	
☐	☒	Antibodies
☐	☒	Eukaryotic cell lines
☒	☐	Palaeontology and archaeology
☐	☒	Animals and other organisms
☒	☐	Clinical data
☒	☐	Dual use research of concern
☒	☐	Plants

Methods

n/a	Involved in the study	
☒	☐	ChIP-seq
☒	☐	Flow cytometry
☒	☐	MRI-based neuroimaging

Antibodies

Antibodies used	In the process of antigenic characterization, six polyclonal sera against A/California/04/2009(H1N1), a triple mutant HA D131E-S193T-A198S, A/California/07/2009(H1N1), A/Utah/20/2009(H1N1), A/Michigan/45/2015(H1N1), and A/Wisconsin/588/2019(H1N1) were utilized. These sera were generated in 4-6 months old influenza seronegative ferrets through nasal infection. The sera used in the study were pooled from two ferrets infected with the same virus.
Validation	To validate the functionality of the ferret antisera, the homologous HAI titers were employed. A ferret antiserum was considered valid if it demonstrated an HAI titer of 1:160 or higher.

Eukaryotic cell lines

Policy information about cell lines and Sex and Gender in Research

Cell line source(s)	Human embryonic kidney (293T) cells and Madin-Darby canine kidney (MDCK) CCL-34 cells () were obtained from the American Type Culture Collection (Manassas, VA).
Authentication	For each cell line, we maintain frozen stocks in liquid nitrogen tanks. Cell lines are replenished about every 20 passages. Cell cultures are inspected daily for sterility, maintenance of adherence, and distinctive morphology. Cell growth media and fetal bovine serum will be tested for quality control and support of cell viability before large-scale purchases are made.
Mycoplasma contamination	Cell lines are regularly screened for mycoplasma contamination. All cell lines were tested negative for mycoplasma contamination
Commonly misidentified lines (See ICLAC register)	No

Animals and other research organisms

Policy information about studies involving animals; ARRIVE guidelines recommended for reporting animal research, and Sex and Gender in Research

Laboratory animals	4-6 month ferrets (Mustela putorius furo)
Wild animals	No
Reporting on sex	No specific sex was excluded during the generation of ferret sera.
Field-collected samples	No
Ethics oversight	Animal study protocols were reviewed and approved by the Institutional Animal Care and Use Committee (IACUC) at Mississippi State University (#14-039) and University of Missouri-Columbia (#38742).

Note that full information on the approval of the study protocol must also be provided in the manuscript.

Plants

Seed stocks	Not applicable
Novel plant genotypes	Not applicable
Authentication	Not applicable
